# Progression of temporal processing deficits in the HIV-1 transgenic rat

**DOI:** 10.1038/srep32831

**Published:** 2016-09-06

**Authors:** Kristen A. McLaurin, Rosemarie M. Booze, Charles F. Mactutus

**Affiliations:** 1 Program in Behavioral Neuroscience Department of Psychology University of South Carolina Columbia, SC 29208, USA.

## Abstract

The HIV-1 transgenic (Tg) rat, which expresses 7 of the 9 HIV-1 genes, was used to investigate the effect(s) of long-term HIV-1 viral protein exposure on chronic neurocognitive deficits observed in pediatric HIV-1 (PHIV). A longitudinal experimental design was used to assess the progression of temporal processing deficits, a potential underlying dimension of neurocognitive impairment in HIV-1. Gap prepulse inhibition (gap-PPI), a translational experimental paradigm, was conducted every thirty days from postnatal day (PD) 30 to PD 180. HIV-1 Tg animals, regardless of sex, displayed profound alterations in the development of temporal processing, assessed using prepulse inhibition. A differential sensitivity to the manipulation of interstimulus interval was observed in HIV-1 Tg animals in comparison to control animals. Moreover, presence of the HIV-1 transgene was diagnosed with 90.8% accuracy using measures of prepulse inhibition and temporal sensitivity. Progression of temporal processing deficits in the HIV-1 Tg rat affords a relatively untapped opportunity to increase our mechanistic understanding of the role of long-term exposure to HIV-1 viral proteins, observed in pediatric HIV-1, in the development of chronic neurological impairment, as well as suggesting an innovative clinical diagnostic screening tool.

Despite the success of combination antiretroviral therapy (cART) in diminishing the prevalence of progressive HIV-1 encephalopathy (PHE), high rates of chronic neurological impairment are still being reported in HIV-1 seropositive children^1^,^2^,^3^,^4^. Approximately 3.2 million children [≤15 years of age^5^] are currently living with HIV-1, however, neurocognitive deficits in HIV-1 seropositive children, including disease progression, are poorly understood^4^. The HIV-1 transgenic (Tg) rat may be used to model the effects of long-term HIV-1 viral protein exposure on the development and progression of chronic neurological deficits observed in children perinatally infected with HIV-1^6^. The HIV-1 Tg rat, originally developed by Reid *et al*.^7^, is non-infectious, expresses 7 of the 9 HIV-1 genes, and exhibits many clinical phenotypes (i.e. cataracts, low body weight) seen in HIV-1 seropositive individuals on cART^8^. Therefore, a longitudinal analysis of temporal processing deficits, a potential underlying dimension of neurocognitive impairment in HIV-1, may provide insight into the effect of long-term HIV-1 viral protein exposure on the development of chronic neurologic impairment in HIV-1 seropositive children.

Deficits in temporal processing, caused by HIV-1, may manifest prior to early symptoms of impairment in higher level cognitive processes^9^,^10^,^11^,^12^. Temporal processing deficits are commonly assessed using translational experimental paradigms, including prepulse inhibition (PPI) of the auditory startle response [ASR; refs 13, 14]. PPI involves two stimuli: a punctate prestimulus or prepulse (i.e., a light or a tone) and a startle stimulus^15^. Presentation of a prepulse 30–500 msec prior to the startle stimulus, causes dramatic reductions in the ASR^15^,^16^. Specifically, the degree to which the ASR is inhibited by the prepulse is dependent upon the interstimulus interval (ISI), the time interval between the prepulse and the startle stimulus. Use of PPI of the ASR, a behavioral approach, may elucidate the underlying mechanisms of more complex neurological processes^15^.

Alterations in temporal processing observed in the HIV-1 Tg rat have been observed in multiple preclinical models of HIV-1, as well as HIV-1 seropositive humans. Specifically, deficits in temporal processing have been observed in Sprague-Dawley rats stereotaxically injected with the HIV-1 viral proteins Tat and/or gp120. Neonatal (postnatal day (PD) 1) intrahippocampal Tat injections flattened the ISI function in auditory PPI, causing a shift in maximum peak inhibition^10^,^17^. A shift in the maximal peak inhibition for auditory PPI was also reported in 9-month old male and female Sprague-Dawley rats neonatally (PD 1) injected with gp120^18^. Temporal processing deficits have also been observed in SIV and FIV positive animals^19^,^20^,^21^,^22^, as well as HIV-1 seropositive individuals meeting criteria for HIV-1 associated neurocognitive disorders (HAND)^11^.

Analyses in the HIV-1 Tg rat have provided additional evidence that temporal processing deficits may be a potential underlying dimension of neurocognitive impairment in HIV-1. Cross-modal PPI was conducted in HIV-1 Tg and control rats between two and six months of age^12^. HIV-1 Tg animals, in comparison to control animals, exhibited significant alterations in the development of temporal processing, evidenced by a relative insensitivity to the manipulation of ISI on both visual and auditory prepulse trials and a lack of perceptual sharpening with age^12^. The generalizability of temporal processing deficits in the HIV-1 Tg rat, assessed using cross-modal PPI, were extended to animals at a more advanced age (i.e., 9, 10 months of age; 23]. HIV-1 Tg animals exhibited a relative insensitivity to the manipulation of ISI in both auditory and visual PPI^23^. Consistent observations of temporal processing deficits regardless of the agent (i.e., single proteins, HIV, SIV, FIV, and the HIV-1 Tg rat) used to study neurocognitive deficits provide ample evidence for the critical importance of understanding the progression of these deficits using a longitudinal experimental design, as was the purpose of the present study.

Lesioning [i.e., ref. 24] and electrical stimulation studies [i.e., refs 25, 26] suggest that PPI of the ASR is mediated through a well-defined serial circuit. Excitatory input from the auditory pathway relays information the inferior colliculus (IC), while input from the visual or tactile pathway relays information to the superior colliculus (SC). From the SC, sensory input subsequently activates the pedunculopontine tegmental nucleus (PPTg), triggering a cholinergic projection to the caudal pontine reticular nucleus (PnC) mediating PPI of the ASR^27^,^28^,^29^,^30^. Activation of the PnC is relayed to motor neurons causing a startle response. Additionally, previous studies have established the hierarchical regulation of PPI, commonly abbreviated as the “CSPP circuitry,” which is comprised of both sequential and parallel neural connections^29^,^30^.

Gap-PPI is another translational experimental paradigm for sensorimotor ‘gating’ that may be used to study temporal processing deficits^31^. Specifically, in gap-PPI the absence of a background stimulus (relative to the presentation of an added stimulus) serves as the punctate prestimulus. Significant reductions in startle amplitude have been observed when a gap in background noise occurs between 30 and 200 msec prior to the startling stimulus^32^. Analyses of gap-PPI in HIV-1 Tg rat at an advanced age (i.e., 9, 10 months of age) revealed a differential sensitivity to the manipulation of ISI. Specifically, control animals exhibited maximal inhibition at the 50 msec ISI, while HIV-1 Tg animals displayed a rightward shift, to maximal inhibition at the 100 msec ISI^23^. Additionally, gap-PPI has been used as an innovative paradigm for studies of tinnitus in animals, including guinea pigs^33^, mice^32^ and rats^34^,^35^,^36^, providing proof-of-concept for the utility of gap-PPI as a translational diagnostic tool for tinnitus in humans. Subsequently, the gap startle paradigm was employed in adult humans with tinnitus offering new insight into the underlying neural mechanisms involved in tinnitus^37^. Use of the gap-PPI experimental paradigms in HIV-1 seropositive children, therefore, may not only serve as a screening tool for chronic neurological impairment in pediatric HIV-1, but may also offer insight into the underlying neural mechanisms implicated in HIV-1.

Modeling the progression of neurocognitive deficits in the HIV-1 Tg rat is fundamental to the advancement of our knowledge of pediatric HIV-1 as well as to the long-term HIV-1 viral protein exposure present in successful cART-treated individuals with HAND. Thus, the aims of the present study were threefold. First, using a longitudinal experimental design, establish the progression of temporal processing deficits using the gap-PPI experimental paradigm in the HIV-1 Tg rat. All animals were tested at 30 day intervals beginning at postnatal day (PD) 30. Second, to investigate the role of sex in the progression of temporal processing deficits in HIV-1; both male and female animals were integral to the experimental design. Thirdly, to assess the ability of gap-PPI to correctly classify animals in regard to their genotype based on measures of prepulse inhibition and temporal sensitivity. Progression of temporal processing deficits in the HIV-1 Tg rat affords a relatively untapped opportunity to increase our mechanistic understanding of the role of long-term exposure to HIV-1 viral proteins, such as seen in pediatric HIV-1 (PHIV), in the development of chronic neurological impairment, as well as suggesting an innovative clinical diagnostic screening tool.

## Methods

Progression of temporal processing deficits in the HIV-1 Tg rat was assessed using a longitudinal analysis of gap-PPI. Alterations in the development of temporal processing were explored using a 2(genotype) x 2(sex) x 6(age) x 6(ISI) x 6(trial) mixed-factor design. A discriminant function analysis was conducted to determine the diagnostic accuracy, and potential translational relevance, of gap-PPI.

### Animals

A longitudinal analysis of gap-PPI was conducted using intact Fischer (F344/N; Harlan Laboratories Inc., Indianapolis, IN) rats (Male HIV-1 Tg, *n* = 26; Female HIV-1 Tg, *n* = 22; Male Control*, n* = 20; Female Control, *n* = 19). Animals were tested for gap-PPI beginning at PD 30 and retested every thirty days at PD 60, PD 90, PD 120, PD 150, and PD 180. Animals were delivered to the facility between PD 7 and PD 9 over the course of four months. All animals were housed with their biological dam until PD 21 when animals were weaned and separated by sex. Subsequently animals were pair- or group-housed with animals of the same sex throughout experimentation. Rodent food (Pro-Lab Rat, Mouse, Hamster Chow #3000) and water were provided *ad libitum* through 60 days of age. Parenthetically, all animals were subsequently under food restriction (85% body weight) due to their participation in a subsequently conducted signal detection task.

Animals were maintained according to the National Institutes of Health (NIH) guidelines in AAALAC-accredited facilities. The targeted environmental conditions for the animal facility were 21° ± 2 °C, 50% ± 10% relative humidity and a 12-h light:12-h dark cycle with lights on at 0700 h (EST). The Institutional Animal Care and Use Committee (IACUC) of the University of South Carolina approved the project protocol as consistent with federal assurance (# A3049-01).

### Apparatus

The startle platform (SR-Lab Startle Reflex System, San Diego Instruments, Inc., San Diego, CA) was enclosed in a 10 cm-thick double-walled, 81 × 81 × 116-cm isolation cabinet (external dimensions) (Industrial Acoustic Company, Inc., Bronx, NY), instead of the 1.9 cm thick ABS plastic or laminate cabinets offered with this system. Sound attenuation of 30 dB (A) was provided in the isolation chamber relative to the external environment. An ambient sound level of 22dB (A) was presented in the chamber without any stimuli presented. The high-frequency loudspeaker of the SR-Lab system (model#40-1278B, Radio Shack, Fort Worth, TX), mounted inside the chamber 30 cm above the Plexiglas animal test cylinder, delivered all auditory stimuli (frequency range of 5k–16 k Hz). The animal’s response to the auditory stimulus produced deflection of the test cylinder, which was converted into analog signals by a piezoelectric accelerometer integral to the bottom of the cylinder. The response signals were digitized (12 bit A to D) and saved to a hard disk. Response sensitivities were calibrated using a SR-LAB Startle Calibration System. Sound levels were measured and calibrated with a sound level meter (model #2203, Bruël & Kjaer, Norcross, GA) with the microphone placed inside the Plexiglas cylinder.

### Gap Prepulse Inhibition Assessments

Intact Fischer rats (Male HIV-1 Tg, *n* = 26; Female HIV-1 Tg, *n* = 22; Male Control*, n* = 20; Female Control, *n* = 19) were tested for gap-PPI of the ASR beginning at PD 30. A 20-min test session began with a 5-min acclimation period in the dark with 70 dB(A) background white noise, followed by six pulse-only ASR trials, used for habituation, with a fixed 10 s intertrial interval (ITI). Thirty-six trials were presented using 6-trial blocks interdigitated using a Latin Square experimental design. Animals were tested with a 20-msec gap in white noise preceding a startle stimulus presented at ISIs of 30, 50,100, and 200 msec. Two control trials, the 0 and 4000 msec ISI trials, were included to provide a reference ASR within gap-PPI. The startle stimulus intensity was 100 dB (A) (20 msec duration) measured inside the test cylinder. Mean peak ASR amplitude values were collected for analysis. All test sessions were conducted in the dark. Because it is well known that many stimulus parameters may influence the dependent measure of startle response amplitude, e.g., duration, intensity, frequency, etc., we choose (and held constant) stimulus parameters for robust prepulse inhibition, but that also would permit detection of increases or decreases in responding. We manipulated the fundamental factor that operationally defines prepulse inhibition, the ISI.

### Statistical Analyses

Data were analyzed using analysis of variance (ANOVA) techniques (SPSS Statistics 20, IBM Corp., Somers, NY). Planned orthogonal contrasts or the post-hoc Greenhouse-Geisser *df* correction factor^38^ were used for potential violations of sphericity of within-subjects variables^39^. Regression analyses and graphs were completed using GraphPad Prism 5 (GraphPad Software, Inc., La Jolla, CA). An alpha level of *p* ≤ 0.05 was considered significant for all statistical tests.

**Figure 1:**
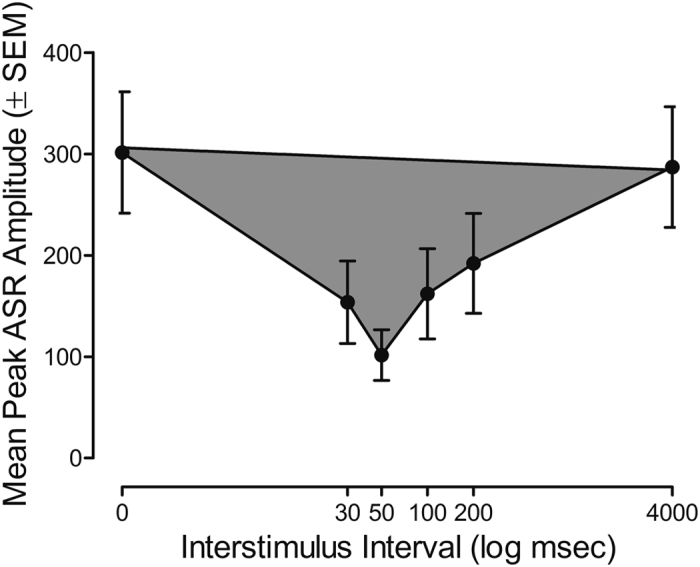
Diagram of the methodology used to derive prepulse inhibition and cumulative frequencies. Prepulse inhibition was derived using area of the peak inflection, which is shaded in gray. The double arrow indicates the potential shift in maximal inhibition, which occurred as a function of age.

The area of inflection of the ASR amplitude response curve, illustrated in Fig. 1 (i.e., shaded gray area), was calculated for each animal at every testing session as a measure of prepulse inhibition. A three-way mixed-factor ANOVA was performed on the mean area of peak inflection where genotype (HIV-1 Tg vs. control) and sex (male vs. female) served as the between-subjects factor. Age served as the within-subjects factor.

The ISI indexing the point of peak inflection for prepulse inhibition, as illustrated in Fig. 1, was also determined for each animal at every testing session. Specifically, a five-way mixed-factor ANOVA was performed on mean peak ASR amplitude for the 0–4000 msec ISIs as a measure of temporal sensitivity. Genotype (HIV-1 Tg vs. control) and sex (male vs. female) served as the between-subjects factor, while age, ISI, and trial served as the within-subjects factors.

The diagnostic accuracy of gap-PPI, i.e., the ability of the observed measures in gap-PPI to correctly identify animals in regard to their genotype (HIV-1 Tg vs. Control) was determined with a discriminant function analysis. Prepulse inhibition measurements (i.e., slope for the area of peak inflection, area of peak inflection at each test session) and temporal sensitivity (i.e., age at which a shift in maximum inhibition was observed) were included in the analysis. Slope for the area of peak inflection was calculated for each animal using the best-fit linear function.

## Results

### HIV-1 Tg rats exhibited a profound alteration in the progression of temporal processing assessed using prepulse inhibition

**Figure 2:**
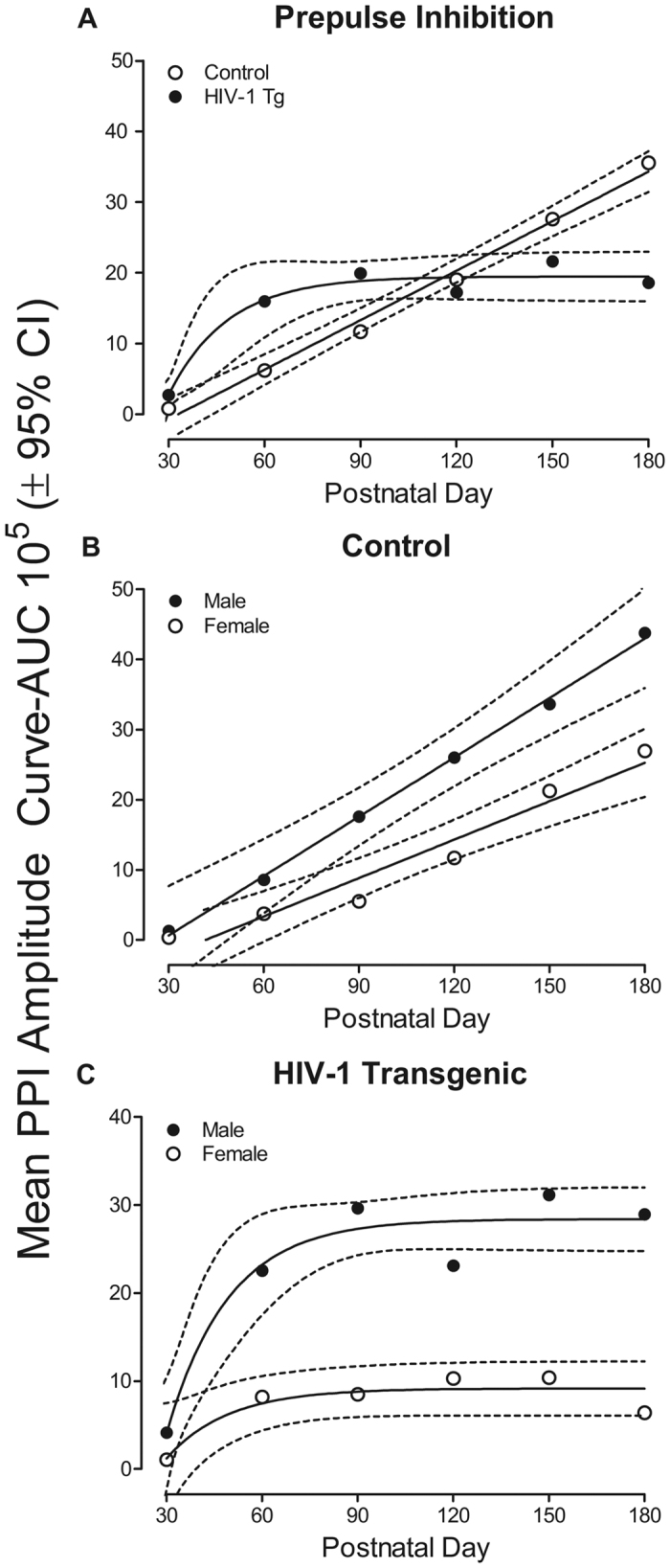
(**a**) Prepulse inhibition, assessed using mean area of the peak inflection, is illustrated as a function of genotype (HIV-1 Tg or Control) and age (±95% CI). As age increases, control animals exhibit an linear increase in prepulse inhibition. Prepulse inhibition for HIV-1 Tg animals, however, was best fit using a one-phase association. Although control animals develop increased prepulse inhibition across age, HIV-1 Tg animals failed to exhibit an increase in prepulse inhibition after PD 90. Linear regression fit (R^2^): HIV-1 Tg, 0.96; Control, 0.99. (**b**) Prepulse inhibition for control animals is illustrated as a function of sex (Male or Female) and age (±95% CI). A first-order polynomial was fit to both male and female control animals. Although a linear increase prepulse inhibition was exhibited by both male and female control animals, there was a significant difference between groups in the rate at which prepulse inhibition increases [F (1,230) = 4.3, *p* ≤ 0.04]. Linear regression fit (R^2^): Male, 0.99; Female, 0.95. (**c**) Prepulse inhibition for HIV-1 Tg animals is illustrated as a function of sex (Male or Female) and age (±95% CI). Prepulse inhibition for both male and female HIV-1 Tg animals was best fit using a one-phase association. Female HIV-1 Tg animals displayed significantly less prepulse inhibition than male HIV-1 Tg animals. Linear regression fit (R^2^): Male, 0.92; Female, 0.82.

The progression of prepulse inhibition, assessed using mean area of the amplitude inflection, was altered in HIV-1 Tg animals in comparison to control animals (Fig. 2a). Prepulse inhibition for control animals was best fit using a first-order polynomial (R^2^: 0.99). In contrast, the best fit for HIV-1 Tg animals was a one-phase association (R^2^: 0.96). Control animals exhibited a significant development of temporal processing across age, while HIV-1 Tg animals failed to exhibit any significant progression of temporal processing after PD 90. The overall ANOVA on mean area of the amplitude inflection confirmed these observations, as revealed by a significant age x genotype interaction [F(5,415) = 6.3, *p*_GG_ ≤ 0.001, η_p_^2^ = 0.07] with a prominent linear component [F(1,83) = 19.0, *p* ≤ 0.001, η_p_^2^ = 0.19] as well as an age x sex interaction [F (5,415) = 2.6, *p*_GG_ ≤ 0.034, η_p_^2^ = 0.03], also with a prominent linear component [F(1,83) = 9.0, *p* ≤ 0.001, η_p_^2^ = 0.10]. Significant main effects of age [F (5,415) = 21.0, *p*_GG_ ≤ 0.001, η_p_^2^ = 0.20] and sex [F (1, 83) = 19.4, *p* ≤ 0.001, η_p_^2^ = 0.19] were also present.

Complementary results were obtained by conducting separate analyses of each genotype, illustrated in Fig. 2b (Control) and 2c (HIV-1 Tg). A first-order polynomial was the best fit for both male and female control animals (Male R^2^: 0.99; Female R^2^: 0.95), indicating an increase in prepulse inhibition across age. However, there was a significant difference in the rate of increase in prepulse inhibition [F(1,230) = 4.3, *p*_GG_ ≤ 0.04]. The rate of temporal processing development was significantly greater in male control animals (β_1_: 2820) in comparison to female control animals (β_1_: 1824). The overall ANOVA for control animals confirmed these observations, as revealed by significant main effects of age [F(5,185) = 20.8, *p*_GG_ ≤ 0.001, η_p_^2^ = 0.36] and sex [F(1,37) = 10.6, *p* ≤ 0.001, η_p_^2^ = 0.22]. In contrast, a one-phase association appeared to be the best fit function for both male and female HIV-1 Tg animals (Male R^2^: 0.92; Female R^2^: 0.82). No significant differences in prepulse inhibition were observed on PD 30. However, at PD 60 and all subsequent test dates, male HIV-1 Tg animals exhibited significantly greater prepulse inhibition in comparison to female HIV-1 Tg animals. The overall ANOVA for HIV-1 Tg animals confirmed these observations, as revealed by significant main effects of age [F(5,230) = 5.8, *p*_GG_ ≤ 0.001, η_p_^2^ = 0.11] and sex [F(1,46) = 11.4, *p* ≤ 0.002, η_p_^2^ = 0.20]. Female HIV-1 Tg animals, therefore, may be more susceptible to the consequences of long-term HIV-1 viral protein exposure in comparison to male HIV-1 Tg animals. However, regardless of sex, prepulse inhibition provided little, if any, compelling evidence for normal development of temporal processing in the HIV-1 Tg rat.

### HIV-1 Tg animals displayed a differential sensitivity to the manipulation of ISI

**Figure 3:**
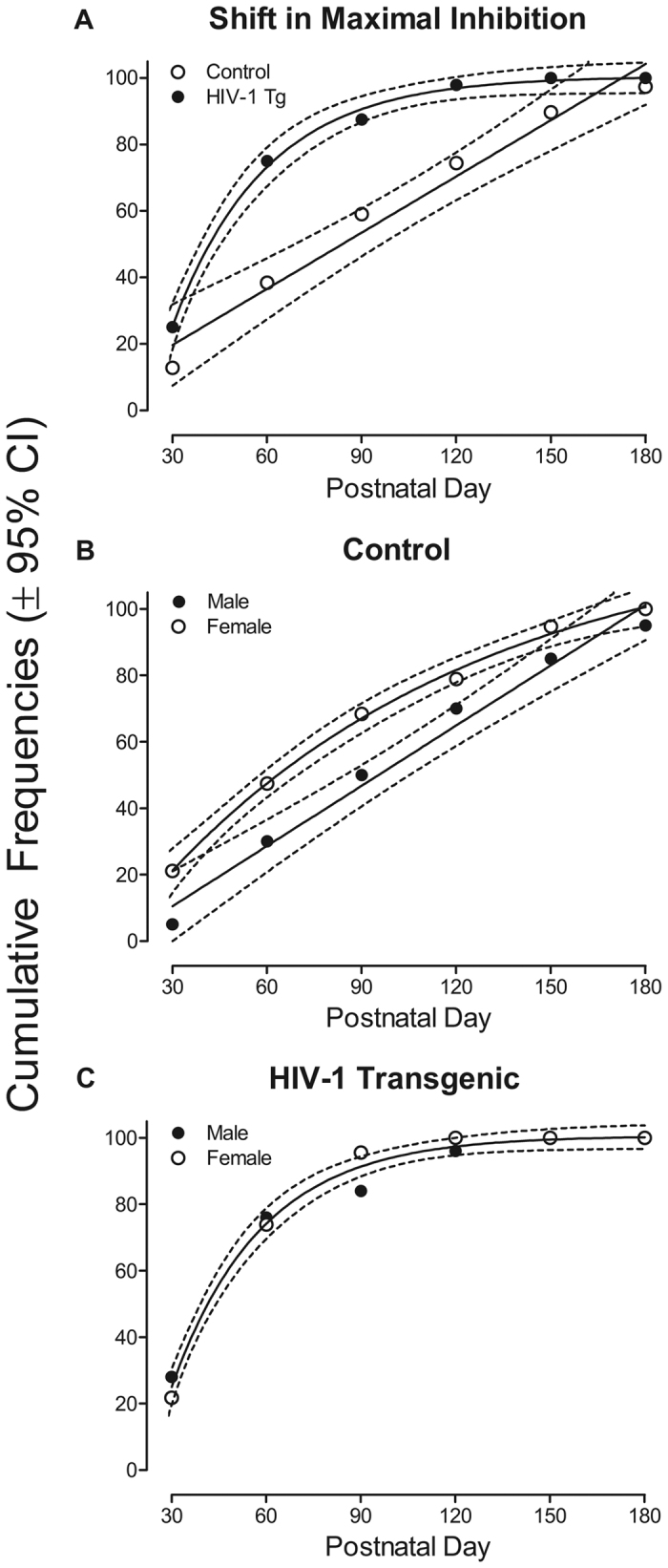
(**a**) The cumulative frequency for the shift in maximal inhibition from 30 msec to 50 msec is illustrated as a function of genotype (HIV-1 Tg or Control) and age (±95% CI). A shift in temporal processing occurred in both the HIV-1 Tg and control animals, however, the shift occurred significantly earlier in the HIV-1 Tg animals. A one-phase association was the best fit for HIV-1 Tg animals, while a first-order polynomial was the best fit for control animals. Linear regression fit (R^2^): HIV-1 Tg, 0.99; Control, 0.97. (**b**) The cumulative frequencies for the shift in maximal inhibition from 30 msec to 50 msec for control animals are illustrated as a function of sex (Male or Female) and age (±95% CI). Female control animals, which were fit using a one-phase association, exhibited a significantly earlier shift in maximal inhibition in comparison to control animals, which were best fit using a first-order polynomial. Linear regression fit (R^2^): Male, 0.98; Female, 0.99. (**c**) The cumulative frequencies for the shift in maximal inhibition from 30 msec to 50 msec for HIV-1 Tg animals are illustrated as a function of sex (Male or Female) and age (±95% CI). A one-phase association global fit was the best fit for both male and female HIV-1 Tg animals. Sex, therefore, did not have a significant effect on sensitivity to the manipulation of ISI in HIV-1 Tg animals. Linear regression fit (R^2^): 0.99.

HIV-1 Tg animals displayed a differential sensitivity to the manipulation of ISI, as illustrated in Fig. 3a. A shift in maximal inhibition (from 30 msec to 50 msec) occurred in both the HIV-1 Tg and control animals; however, the shift occurred significantly earlier in the HIV-1 Tg animals. In the control group, a first-order polynomial (R^2^: 0.97) suggested peak inhibition was observed at the 30 msec ISI during the first three test periods (PD 30, PD 60 and PD 90), but that rightward shift to the 50 msec ISI occurred during the latter three test periods (PD 120, PD 150 and PD 180). In contrast, the HIV-1 Tg group, well-described with a one-phase association (R^2^: 0.99), displayed peak inhibition at 30 msec during the first testing period (PD 30), but displayed a rightward shift during all later testing periods. The overall ANOVA revealed significant three-way interactions: genotype x age x ISI interaction [F(25,2050) = 11.1, *p*_GG_ ≤ 0.001, η_p_^2^ = 0.12] and genotype x age x sex interaction [F(5,410) = 4.5, *p*_GG_ ≤ 0.001, η_p_^2^ = 0.05], and an age x ISI x sex interaction [F(25,2050) = 4.4, *p*_GG_ ≤ 0.001, η_p_^2^ = 0.05]. Second order interactions were also confirmed including: a genotype x age interaction [F(5,410) = 54.4, *p*_GG_ ≤ 0.001, η_p_^2^ = 0.40], an age x ISI interaction [F(25,2050) = 26.3, *p*_GG_ ≤ 0.01, η_p_^2^ = 0.24], an age x sex interaction [F(5,410) = 10.2, *p*_GG_ ≤ 0.001, η_p_^2^ = 0.11], an ISI x genotype interaction [F(5,410) = 7.1, *p*_GG_ ≤ 0.001, η_p_^2^ = 0.08] and an ISI x sex interaction [F(5,410) = 23.3, *p*_GG_ ≤ 0.001, η_p_^2^ = 0.22]. Significant main effects of age [F(5,410) = 68.3, *p*_GG_ ≤ 0.001, η_p_^2^ = 0.45], ISI [F(5,410) = 164.9, *p*_GG_ ≤ 0.001, η_p_^2^ = 0.67], genotype [F(1,82) = 100.7, *p* ≤ 0.001, η_p_^2^ = 0.55], and sex [F(1,82) = 40.6, *p* ≤ 0.001, η_p_^2^ = 0.33] were also noted. Clearly, there was a significantly greater effect of age on the variation of response amplitude by ISI for HIV-1 Tg animals in comparison to control animals.

Analyses of each genotype were conducted to further examine differences in the progression of temporal processing (Control: Fig. 3b; HIV-1 Tg: Fig. 3c). A first-order polynomial was a well-described fit for male control animals (R^2^: 0.98), while a one-phase association provided a well-described fit for female control animals (R^2^: 0.99). Specifically, female control animals exhibited a shift in maximal inhibition from 30 msec to 50 msec significantly earlier than male control animals. The overall ANOVA for control animals revealed an age x ISI x sex interaction [F(25,900) = 2.9, *p*_GG_ ≤ 0.001, η_p_^2^ = 0.08], an age x ISI interaction [F (25,900) = 23.3, *p*_GG_ ≤ 0.001, η_p_^2^ = 0.39], an age x sex interaction [F(5,180) = 7.2, *p*_GG_ ≤ 0.001, η_p_^2^ = 0.17], and an ISI x sex interaction [F(5,180) = 12.6, *p*_GG_ ≤ 0.001, η_p_^2^ = 0.26]. A significant main effect of age [F (5,180) = 62.8, *p*_GG_ ≤ 0.001, η_p_^2^ = 0.64], ISI [F (5,180) = 111.4, *p*_GG_ ≤ 0.001, η_p_^2^ = 0.76], and sex [F(1,36) = 16.0, *p* ≤ 0.001, η_p_^2^ = 0.31] were also revealed. In contrast, a one-phase association provided a well-described fit for both female and male HIV-1 Tg animals (R^2^: 0.99) suggesting that the shift in maximal inhibition from 30 msec to 50 msec was not dependent upon sex in the presence of the HIV-1 transgene. The overall ANOVA for the HIV-1 Tg group did not reveal a significant age x sex interaction [F(5,230) = 1.7, *p*_GG_ ≤ 0.146, η_p_^2^ = 0.04], nor a main effect of age [F (5,230) = 1.4, *p*_GG_ ≤ 0.246, η_p_^2^ = 0.03], confirming these observations. Most notably, therefore, regardless of sex, HIV-1 Tg animals exhibited a significantly earlier shift in maximal inhibition, indicating a differential sensitivity to the manipulation of ISI.

### Longitudinal gap-PPI can accurately diagnose the presence of the HIV-1 Transgene

**Figure 4:**
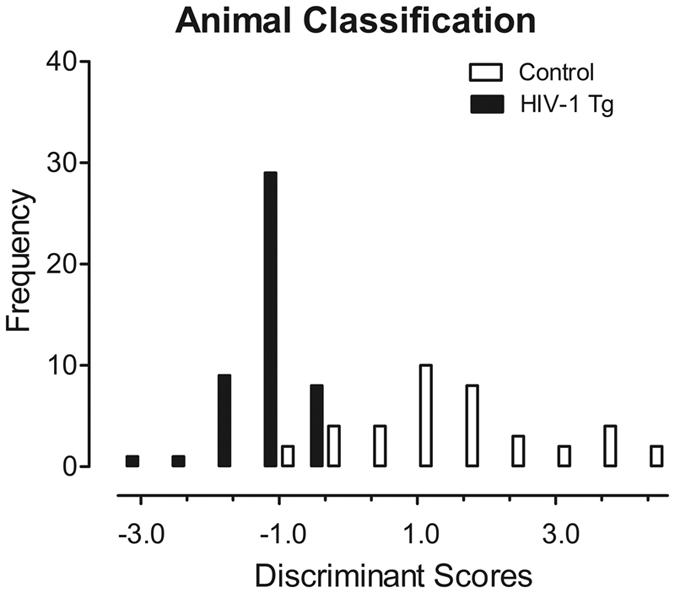
Animal classification is illustrated as a function of the canonical variable representing the simplest linear function that best separated the HIV-1 Tg and control groups (canonical correlation 0.80) and correctly identified (jackknife classification) group membership with 90.8% accuracy (79.5% of controls, and 100% of HIV-1 Tg animals).

Progression of temporal processing, assessed using gap-PPI, was further analyzed using a discriminant function analysis to determine the potential utility of gap-PPI as a diagnostic screening tool for chronic neurological impairment associated with the HIV-1 transgene. Transgene presence was best predicted using measures of prepulse inhibition (i.e., mean area of the peak inflection) and measures of temporal sensitivity (i.e., cumulative frequency), as illustrated in Fig. 4. Specifically, the discriminant function analysis selected four variables (slope of area of the peak inflection, area of the peak inflection at PD 180 and cumulative frequency at PD 60 and PD 180) that maximally separated the HIV-1 Tg and control animals (canonical correlation of 0.80). Animals were correctly diagnosed for the presence of the HIV-1 transgene with 90.8% accuracy (F approximation of Wilks’ λ of 0.368, F (4, 82) = 35.2, *p* ≤ 0.001).

## Discussion

Prominent temporal processing deficits in the HIV-1 Tg rat were detected using the gap-PPI experimental paradigm. The developmental trajectory of prepulse inhibition, assessed using the gap-PPI experimental paradigm, was altered in the HIV-1 Tg rat relative to control animals, regardless of sex, from 30 to 180 days of age. Additionally, female HIV-1 Tg animals exhibited significantly less prepulse inhibition in comparison to male HIV-1 Tg animals. A differential sensitivity to the manipulation of ISI was also observed in the HIV-1 Tg rat, regardless of sex. Animals were correctly classified (90.8%) for the presence of the genotype (HIV-1 Tg or control) using measures of prepulse inhibition and temporal sensitivity. Temporal processing deficits observed in the HIV-1 Tg rat resemble those reported in HIV-1 seropositive individuals, providing insight into the effect of long-term exposure to HIV-1 viral proteins and the potential for development of a diagnostic screening tool for chronic neurological impairment.

HIV-1 Tg rats displayed a differential development of prepulse inhibition, assessed using the gap-PPI experimental paradigm, in a longitudinal experimental design. A one-phase association was the best fit for HIV-1 Tg animals, while a first-order polynomial was the best fit for control animals. No statistical differences in temporal processing between the HIV-1 Tg and control groups were observed at PD 30. However, significant differences in the progression of temporal processing, assessed using prepulse inhibition, were exhibited at subsequent testing sessions. Specifically, control animals displayed a linear increase in prepulse inhibition from PD 30 to PD 180. In contrast, HIV-1 Tg animals only exhibited a significant increase in prepulse inhibition from PD 30 to PD 90, after which point a plateau in temporal processing was observed. To our knowledge, the present study is the first to investigate the progression of temporal processing deficits using gap-PPI in the HIV-1 Tg rat, which is vital for the development of an extant model for HAND.

Sex differences in the progression of temporal processing deficits, assessed using prepulse inhibition, provide fundamental information for our understanding of the long-term effect of HIV-1 viral protein exposure in pediatric HIV-1. Both female HIV-1 Tg and control animals displayed significantly less prepulse inhibition, in comparison to male HIV-1 Tg and control animals, consistent with previous observations in healthy individuals^40^,^41^,^42^. Regardless of sex, however, HIV-1 Tg animals exhibited significant alterations in temporal processing, assessed using prepulse inhibition; alterations which were more profound in female HIV-1 Tg animals in comparison to male HIV-1 Tg animals. Sex differences in the HIV-1 Tg rat expand on differences observed in HIV-1 seropositive individuals. Recent studies have reported significantly greater neurological impairment in HIV-1 seropositive women, in comparison to HIV-1 seropositive men^43^,^44^. The effect of sex on the progression of neurocognitive impairments in HIV-1 seropositive individuals has been relatively understudied; the need for longitudinal study is critical^45^.

A differential sensitivity to the manipulation of ISI was evidenced by a shift in the point of maximal inhibition. Both HIV-1 Tg and control animals displayed maximal inhibition at the 30 msec ISI on PD 30. However, HIV-1 Tg animals displayed a rightward shift in peak inhibition, from 30 msec to 50 msec, at PD 60. Notably, no sex differences were observed in the HIV-1 Tg animals. The point of maximal peak inhibition in control animals shifted from 30 msec to 50 msec at PD 120. Female control animals exhibited a significantly earlier shift in maximal inhibition in comparison to male control animals. Results of differential sensitivity to the manipulation of ISI in the present study extend those previously reported in the HIV-1 Tg rat^12^,^23^ and Sprague-Dawley rats stereotaxically injected with the HIV-1 viral proteins^10^,^17^,^18^.

Temporal processing deficits observed using gap-PPI in the HIV-1 Tg rat may provide a novel screening tool for the diagnosis of chronic neurological impairment in pediatric HIV-1. A discriminant function analysis, used to evaluate the potential utility of gap-PPI, correctly identified animals in regards to their genotype (HIV-1 Tg vs. control) with 90.8% accuracy. The presence of the HIV-1 transgene was best predicted using four variables as assessments of prepulse inhibition (i.e., slope of area of the peak inflection, area of the peak inflection at PD 180) and temporal sensitivity (i.e., cumulative frequency at PD 60 and PD 180). Gap-PPI was previously implicated as a novel screening tool for the diagnosis of tinnitus. Specifically, rats with noise over-expression-induced tinnitus^33^ or salicylate-induced tinnitus^36^ exhibited little to no inhibition in gap-PPI studies. The gap-PPI experimental paradigm was subsequently employed in adult humans with tinnitus offering new insight into the neural mechanisms of tinnitus^37^. Use of the gap-PPI experimental paradigms in HIV-1 seropositive humans, therefore, may not only serve as a screening tool for chronic neurological impairment in pediatric HIV-1, but may also offer insight into the underlying neural mechanisms implicated in HAND.

Clinical and preclinical studies have implicated disruptions in the development of the dopamine (DA) system as an underlying factor in neurocognitive impairments observed in pediatric HIV-1^46^,^47^,^48^,^49^,^50^. A case study of five HIV-1 seropositive children observed improved motor function and activity with administration of levodopa (L-DOPA), a DA agonist^51^. In addition, pharmacological assessments have previously been used to examine alterations in the midbrain dopaminergic system in the HIV-1 Tg rat^48^,^50^. Dopaminergic system dysfunction, assessed using Western blotting, was evidenced by alterations in phosphorylated tyrosine hydroxylase (pTH), dopamine transporter (DAT) mRNA, and/or monoamine oxidase A [MAO-A; 48; 50]. In addition, young HIV-1 Tg rats exhibit deficits in D_2_/_3_ receptors in the dorsal striatum, assessed using [^18^F]fallypride positron emission tomography (PET)^47^.

DA system dysfunction may underlie temporal processing deficits observed in the HIV-1 Tg rat, as evidenced by previous behavioral and pharmacological studies^52^,^53^,^54^. Administration of direct (i.e., apomorphine) and indirect DA agonists (i.e., amphetamine) have been used to manipulate dopamine, subsequently disrupting PPI^52^. We have previously observed an insensitivity to the manipulation of ISI, assessed using cross-modal PPI, in rats administered apomorphine; results that are comparable to those observed in the HIV-1 Tg rat^53^ and schizophrenic patients^55^,^56^. Although temporal processing deficits observed in the HIV-1 Tg rat may be mediated by multiple neural systems, HIV-1 infection often affects DA system function and is associated with subsequent cognitive deficits^57^,^58^.

The potential role of oxidative stress and neuroinflammation as well as synaptodendritic injury are other potential mechanisms contributing to neurocognitive impairment in the HIV-1 Tg rat. Prior research has reported evidence of chronic immune activation in the brains of the HIV-1 Tg rat^59^,^60^. Thus, in the absence of viral replication, a chronic, mild, neuroinflammatory microenvironment exists in the HIV-1 Tg animals. Interestingly, the dopaminergic system appears to be particularly sensitive to neuroinflammatory degenerative processes^61^,^62^. Decifits in synaptic proteins as well as decreases in dendritic branching complexity and shifts in dendritic spine parameters have also been established^59^,^63^. HIV-1 associated neurocognitive disorders are thought to be the result of dendritic injury^64^ and synaptic dysfunction^65^.

The interpretation of the results presented herein and the longitudinal experimental design employed deserves further consideration. Prior longitudinal assessments of temporal processing provide evidence for experienced-based improvements^66^,^67^,^68^, a potential caveat for interpretation of the present study. However, assessments of gap-PPI in the present study began at PD 30, after the ontogeny of gap-PPI attains mature level (PD 28)^66^,^69^. Although the response amplitude per se may decrease as a function of experience^66^,^67^,^68^, there were no significant shifts in the ISI for peak inhibition in the amplitude response curve, which are observed in the present study as a function of age (i.e., Fig. 3). Critically, all animals in the present study were subjected to the same experiences during early development and immediately prior to experimentation; a level of control not present in studies with humans. As previously observed^12^, the protracted development of perceptual sharpening across repeated testing, as indexed by the relative insensitivity to ISI, may reflect, in part, impaired CNS plasticity in the HIV-1 Tg rat.

The use of the classical ISI approach in the gap-PPI experimental paradigm also merits further discussion. The classic approach, popularized by Ison and Hammond^14^, relies on the manipulation of the ISI as the fundamental operationally defined factor for PPI to assess sensorimotor ‘gating’, i.e., a measure of temporal processing. The present study employs a constant gap duration (i.e., 20 msec) and a range of ISI values to accurately assess the response amplitude curves for gap-PPI. Temporal acuity per se, as may be assessed via manipulation of the stimulus parameter of gap duration^66^,^67^,^68^, was not measured in the present study. Braff *et al*.^56^ assessed the effect of re-test on the startle reflex employing the classical ISI approach. Startle response is a relatively stable phenomenon when the classical ISI approach is employed^56^, providing additional evidence that the present results are not an effect of experience. Thus, the use of a longitudinal experimental design and manipulation of ISI in gap-PPI affords an undeniable opportunity to assess the effect of age on the development of perceptual sharpening, of temporal sensitivity, which may be a critical underlying dimension for the cognitive impairments observed in HAND.

The study of early neurological deficits is vital for the development of an extant model for the progression of neurocognitive deficits in HAND, as well as the development of an effective treatment for pediatric HIV-1. The HIV-1 Tg rat, which resembles HIV-1 seropositive individuals on cART, provides a vehicle for understanding the progression of neurological impairment using a longitudinal experimental design. HIV-1 Tg rats used in the present study displayed no significant health disparities in comparison to F344 controls (i.e., similar growth rates), consistent with our previous studies^12^,^63^,^70^. HIV-1 Tg animals used in the current study are a healthier derivation of those originally described^7^, which displayed severe phenotypical alterations (e.g., hindlimb paralysis), and wasting at a relatively young age (5–9 months). In contrast, no general wasting or pathological phenotypes were observed in the HIV-1 Tg animals used in the current study. Both HIV-1 Tg and control animals display similar inhibition in visual PPI suggesting that, despite the presence of cataracts in the HIV-1 Tg group, animals were able to detect the 20 msec visual stimulus presented^12^. Thus, the current HIV-1 Tg rat displays a moderate phenotype closely resembling HIV-1 seropositive children on cART, making it useful for longitudinal studies of HAND progression.

Temporal processing deficits observed in the HIV-1 Tg rat, assessed using gap-PPI, resemble temporal processing deficits commonly exhibited in HIV-1 seropositive individuals. Understanding the altered progression of temporal processing and differential sensitivity to the manipulation of ISI fills previous gaps in our knowledge about the effect of long-term exposure to HIV-1 viral proteins on neurocognitive impairment evident in pediatric HIV-1 and may reveal information regarding the underlying neural circuitry. Temporal processing measures suggest a diagnostic screening tool for chronic neurological impairment, as well as a venue for the development of novel therapeutics.

## Additional Information

**How to cite this article**: McLaurin, K. A. *et al*. Progression of temporal processing deficits in the HIV-1 transgenic rat. *Sci. Rep.* **6**, 32831; doi: 10.1038/srep32831 (2016).
